# PbSR is synthesized in macrogametocytes and involved in formation of the malaria crystalloids

**DOI:** 10.1111/j.1365-2958.2008.06254.x

**Published:** 2008-04-29

**Authors:** Victoria Carter, Shoichi Shimizu, Meiji Arai, Johannes T Dessens

**Affiliations:** 1 Department of Infectious and Tropical Diseases, London School of Hygiene and Tropical MedicineKeppel Street, London WC1E 7HT, UK; 2 Department of Immunology and Parasitology, University of Occupational and Environmental HealthJapan, 1-1 Iseigaoka, Yahatanishi-ku, Kitakyusyu, Fukuoka 807-8555, Japan

## Abstract

Crystalloids are transient organelles that form in developing malaria ookinetes and disappear after ookinete-to-oocyst transition. Their origins and functions remain poorly understood. The *Plasmodium berghei* scavenger receptor-like protein PbSR is essential for mosquito-to-host transmission of the parasite: PbSR knockout parasites produce normal numbers of oocysts that fail to form sporozoites, pointing to a role for PbSR in the oocyst during sporogony. Here, using fluorescent protein tagging and targeted gene disruption, we show that PbSR is synthesized in macrogametocytes, gets targeted to the crystalloids of developing ookinetes and is involved in crystalloid formation. While oocyst sporulation rates of PbSR knockout parasites are highly reduced in parasite-infected mosquitoes, sporulation rates *in vitro* are not adversely affected, supporting the view that mosquito factors could be involved in the PbSR loss-of-function phenotype. These findings are the first to identify a parasite protein involved with the crystalloid organelle, and suggest a novel protein-trafficking mechanism to deliver PbSR to the oocysts.

## Introduction

Malaria is the most important parasitic infection in people and causes over one million deaths annually (Greenwood *et al*., 2005). Malaria control efforts suffer from widespread resistance to antiparasitic drugs and insecticides, underpinning the urgent need for novel intervention strategies. Transmission of malaria parasites starts with the ingestion of female (macro-) and male (micro-)gametocytes by vector mosquitoes during blood feeding on a parasite-infected host. Rapid gametogenesis and fertilization occur in the mosquito midgut, from which ookinetes develop that traverse the midgut epithelium and transform into oocysts. After a 2-week period of growth, mature oocysts release thousands of sporozoites into the mosquito haemolymph that invade the salivary glands of the insect and enter the vertebrate host during blood feeding to initiate new malaria infections.

A unique parasite organelle, the crystalloid, is formed during ookinete development. *Plasmodium* crystalloids are cytoplasmic aggregations of closely packed spherical particles 25–35 nm in diameter (Garnham *et al*., 1962; 1969). Because of their resemblance to viral inclusions it has been postulated that the crystalloids could be of viral origin (DasGupta, 1968; Terzakis, 1969; Terzakis *et al*., 1976). The fact that crystalloids disappear during the early stages of oocyst growth has led others to postulate that these structures could constitute a reservoir of protein synthesized by the macrogametocyte that is used by the parasite during oocyst development (Garnham, 1966; 1969; Trefiak and Desser, 1973). However, no experimental data have been produced to substantiate these hypotheses, and hence the origins and functions of this organelle have remained elusive for more that 40 years.

The rodent malaria *Plasmodium berghei* scavenger receptor-like protein PbSR (also called PbLAP1) is a modular protein containing several distinct adhesive-type domains implicated in lipid, protein and carbohydrate binding. It is highly conserved among *Plasmodium* species. In addition to its adhesive domains, the presence of an ER signal peptide and the absence of typical organellar-targeting sequences suggested an extracellular role for PbSR, possibly involving interaction with the vertebrate host or insect vector (Claudianos *et al*., 2002; Dessens *et al*., 2004). It was previously shown that PbSR is expressed in sporozoites, and that genetically modified PbSR knockout parasites form normal numbers of oocysts in vector mosquitoes, but fail to produce sporozoites (Claudianos *et al*., 2002). While these results pointed to a role of PbSR in the oocyst during sporogony, the mode of action of this molecule has remained speculative. PbSR contains two scavenger receptor cysteine-rich (SRCR) domains that most closely resemble those found in proteins involved in recognition and activation of the immune system in the mouse (Spa, CD5, CD6, CD163), fruit fly (GRAAL/Sp22D) and sea urchin (SpSRCR7) (Claudianos *et al*., 2002). In addition, PbSR contains a pentaxin domain that is shared with several proteins, including complement-reactive protein and serum amyloid protein, which are involved in acute immunological responses in vertebrates. Based on these structural homologies with established immune-type metazoan proteins, PbSR has been postulated to play a role in the evasion of, or the protection from, immune factors in the insect or vertebrate host (Claudianos *et al*., 2002; Delrieu *et al*., 2002; Lasonder *et al*., 2002).

In this study we monitored the development of PbSR knockout parasites *in vitro* and show that under culture conditions oocyst sporulation rates are comparable to those of parasites expressing functional PbSR, supporting the view that the function of PbSR involves interaction with mosquito factors. Furthermore, by examining the expression and subcellular localization of PbSR using fluorescent protein tagging, we show that PbSR is not expressed in sporozoites as shown previously (Claudianos *et al*., 2002; Trueman *et al*., 2004), but instead is synthesized in blood-stage macrogametocytes. Our data further show that PbSR is targeted to the crystalloids and is involved in crystalloid formation. The biological significance of these results in relation to PbSR expression, trafficking and function is discussed.

## Results

### Generation and molecular analyses of genetically modified parasite lines

**Fig. 1:**
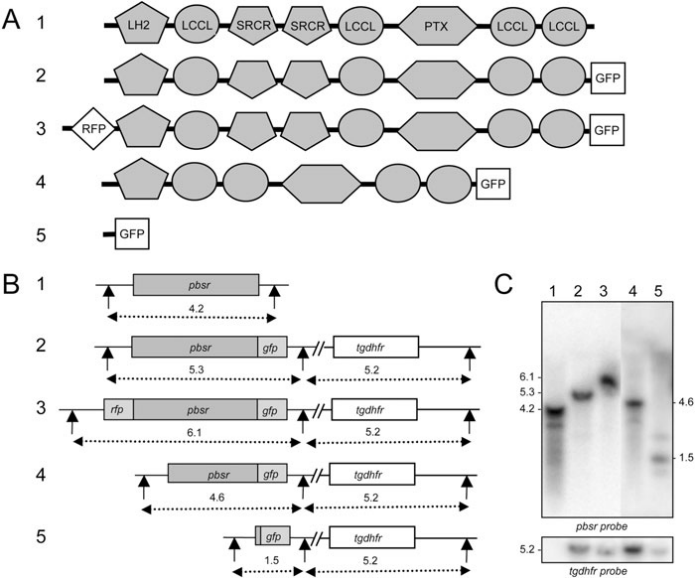
Generation and molecular analysis of genetically modified parasite lines. A. Schematic diagram of the different versions of PbSR expressed by the parasite lines used. 1, wild-type parental parasite; 2, PbSR/EGFP; 3, mCherry/PbSR/EGFP; 4, ΔSRCR/EGFP; 5, ΔPbSR/EGFP. Indicated are the lipoxygenase homology domain (LH2), the *Limulus* clotting factor C, Coch-5b2, Lgl1 domain (LCCL); the scavenger receptor cysteine-rich domain (SRCR), the pentaxin domain (PTX), the green fluorescent protein module (GFP) and the red fluorescent protein module (RFP). B. Schematic diagram of the corresponding wild-type and genetically modified *pbsr* loci on genomic DNA. Numbers 1–5 correspond to the parasite lines shown in (A). Indicated are positions of the SpeI restriction sites (vertical arrows), and expected SpeI restriction fragments (dotted lines) with sizes shown in kb. C. Southern blot analysis of SpeI-digested parasite genomic DNA. Lanes 1–5 correspond to the parasite lines shown in (A). Indicated are the sizes of the *pbsr*- and *tgdhfr*-specific fragments.

A series of genetically modified parasite lines was generated (Fig. 1A) to assess PbSR expression and function. This was achieved by stably replacing the native *pbsr* locus with recombinant, fluorescent protein-tagged versions of the *pbsr* gene, using double-cross-over homologous recombination (van Dijk *et al*., 1995; Waters *et al*., 1997). Concomitantly, a modified *Toxoplasma gondii* dihydrofolate reductase (*tgdhfr*) gene cassette was introduced conferring resistance against the antimalarial drug pyrimethamine (Fig. 1B). After transfection of purified schizont preparations, pyrimethamine resistant parasites were selected. Diagnostic PCR across the predicted integration sites showed correct integration of the *tgdhfr* cassette into the *pbsr* locus (data not shown). This was confirmed by assessing the integrity of clonal populations of the genetically modified parasite lines by Southern blot analysis of SpeI-digested genomic DNA (Fig. 1C). Accordingly, a probe corresponding to the 5′UTR plus coding sequence of *pbsr* gave rise to an expected band of 4.2 kb in the parental, wild-type parasite. In agreement with the genetic modifications, the size of this fragment changed to 5.3 kb, 6.1 kb, 4.6 kb and 1.5 kb, respectively, in parasite lines PbSR/EGFP, mCherry/PbSR/EGFP, ΔSRCR/EGFP and ΔPbSR/EGFP (Fig. 1C). Conversely, a probe corresponding to the *tgdhfr* gene gave rise to an expected band of 5.2 kb in the genetically modified parasite lines, and no signal in wild-type parasites (Fig. 1C). These combined results demonstrate correct integration of the recombinant *pbsr* and selectable marker genes into the native *pbsr* locus. All genetically modified clonal parasite lines developed normally in mice and were morphologically indistinguishable from wild-type parasites in Giemsa-stained blood films (data not shown).

### Life stage expression of PbSR

We initially assessed PbSR protein expression in parasite line PbSR/EGFP, which expresses the full-length PbSR protein containing a C-terminal green fluorescent protein (GFP) tag (Fig. 1). GFP fluorescence was observed in a subpopulation of parasitized erythrocytes. Upon gametocyte activation, the majority of fluorescent parasites emerged from the host cell (visible by the rounding up of the parasite), indicating that this subpopulation consisted of gametocytes. Exflagellation (the release of male gametes) was only observed in gametocytes that were not green, indicating that PbSR expression was restricted to female gametocytes. Parasitized blood that was immediately fixed in paraformaldehyde also contained GFP-based fluorescent parasites, demonstrating that GFP expression was not the result of gametocyte activation. These combined observations indicate that within the blood-stage parasite population, PbSR is predominantly expressed in female gametocytes. This is in full agreement with results of a *pbsr* promoter-driven reporter study (Khan *et al*., 2005).

**Fig. 2:**
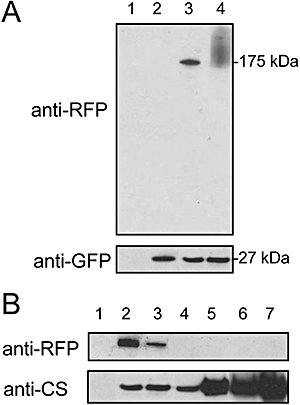
Life stage expression of PbSR by Western blot analysis. A. Purified gametocytes from wild-type (lane 1), PbSR/EGFP (lane 2) and mCherry/PbSR/EGFP (lanes 3 and 4) parasites under reducing (lanes 1–3) and non-reducing (lane 4) conditions. Blot was developed with anti-RFP (top) or anti-GFP (bottom) antibodies. B. Western blot analysis of different mCherry/PbSR/EGFP life stages: purified asexual blood stages (lane 1); purified gametocytes (lane 2); purified ookinetes (lane 3); two heavily infected midguts at 5 days post infection (lane 4); two heavily infected midguts at 11 days post infection (lane 5); purified midgut sporozoites (lane 6); purified salivary gland sporozoites (lane 7). Blot was developed with anti-RFP (top) or anti-CS (bottom) antibodies.

Analysis of purified PbSR/EGFP gametocytes by Western blot using an anti-GFP antibody failed to detect the full-length PbSR/GFP product (predicted to be 175 kDa in size), but gave rise to a band of 27 kDa corresponding to monomeric GFP (Fig. 2A). This indicated that the C-terminal GFP tag was efficiently cleaved off the full-length PbSR protein. For this reason we generated a second parasite line (mCherry/PbSR/EGFP) which, in addition to the C-terminal GFP tag, contained an N-terminal red fluorescent protein (RFP) tag (Fig. 1). This parasite line produced female gametocytes that were both red and green fluorescent. Western blot analysis revealed that the C-terminal GFP tag was again efficiently cleaved off the expressed PbSR protein (Fig. 2A). However, anti-RFP antibody detected full-length RFP/PbSR protein (also predicted to be 175 kDa in size) (Fig. 2A), showing that the N-terminal RFP tag remains intact. Under non-reducing conditions the RFP/PbSR protein migrated as a smear of high-molecular-weight bands (Fig. 2A), indicating that PbSR forms protein complexes through disulphide bond formation.

Both parasite lines PbSR/EGFP and mCherry/PbSR/EGFP gave rise to large numbers of sporozoites in parasite-infected mosquitoes, and were efficiently transmitted to naïve mice by infected mosquito bites (data not shown), demonstrating that the fluorescent protein tags did not affect functionality of the PbSR protein. To assess PbSR protein expression in different life stages, we analysed parasite line mCherry/PbSR/EGFP by western blot with anti-RFP antibody. This analysis confirmed that PbSR is present in gametocytes and is also present, at lower levels, in ookinetes (Fig. 2B). However, PbSR was not detected in asexual blood stages, oocysts and sporozoites with this assay (Fig. 2B). Circumsporozoite protein (CS), by comparison, was present in all life stages examined except asexual blood stages (Fig. 2B), in accordance with its described life stage expression pattern (Natarajan *et al*., 2001).

### Subcellular localization of PbSR

**Fig. 3:**
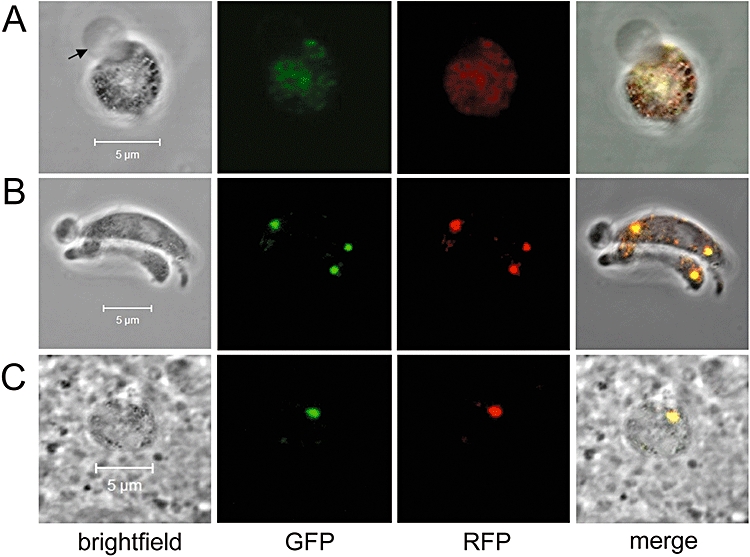
Cellular localization of PbSR by fluorescence confocal microscopy. A. Female gametocyte just emerged from the host erythrocyte, whose membrane is still attached (arrow). B. Group of three mature ookinetes. C. Very young oocyst/rounded-up ookinete situated on the basal side of a midgut at 2 days post mosquito infection.

Confocal microscopic examination of mCherry/PbSR/EGFP parasites revealed green and red fluorescence throughout the cytoplasm of female gametocytes/macrogametes (Fig. 3A). This localization was not obviously associated with the periphery of the parasites, or with their host erythrocytes. A similar fluorescence pattern was observed in zygotes and early retorts (i.e. immature ookinetes) (data not shown). In contrast, in mature ookinetes fluorescence had become condensed into one to two cytoplasmic focal spots (Fig. 3B). Similar spots were observed in young oocysts on the basal side of mosquito midguts for up to 2 days post mosquito infection (Fig. 3C). Asexual stages, male gametocytes, microgametes, late oocysts and sporozoites were negative for both GFP and RFP fluorescence (data not shown). Red and green fluorescence colocalized in PbSR-positive cells (Fig. 3), suggesting that the GFP tag remains associated with the PbSR protein complex despite being cleaved. Consistent with this observation, PbSR/EGFP parasites displayed the same fluorescence pattern as mCherry/PbSR/EGFP parasites (data not shown).

**Fig. 4:**
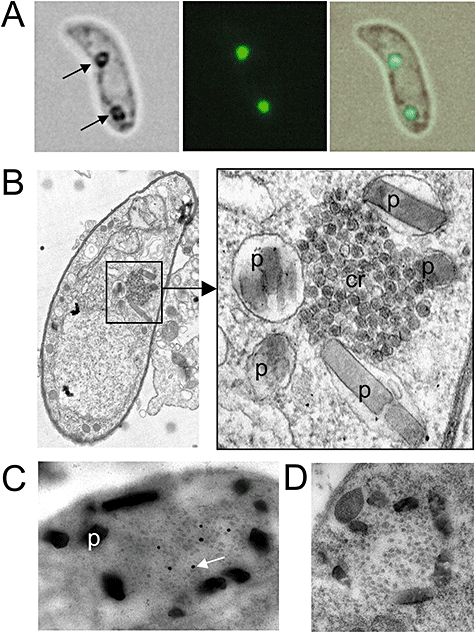
PbSR is targeted to the crystalloid organelle of the ookinete. A. Image of an ookinete showing the colocalization of the fluorescent spots with malaria pigment (arrows). B. Transmission electron microscopic image of a whole ookinete section, and a subsection thereof at higher magnification (boxed) showing the crystalloid (cr) surrounded by vacuoles containing malaria pigment (p). C. Immunogold labelling (arrow) of the ookinete crystalloid in parasite line mCherry/PbSR/EGFP. This picture again clearly shows how the crystalloid is surrounded by pigment (p). D. Immunogold electron micrograph of a wild-type ookinete, showing absence of labelling.

To determine the origin of the focal spots observed in mCherry/PbSR/EGFP and PbSR/EGFP ookinetes we initially tested for mitochondrial and lysozomal targeting using Mitotracker and Lysotracker, respectively, but found no colocalization with the fluorescent spots (data not shown), indicating that PbSR was targeted neither to mitochondrial nor to lysosomal organelles. Closer examination of these ookinetes, however, revealed a clear colocalization of the fluorescence with malaria pigment (Fig. 4A). Malaria pigment, or haemozoin, is formed as a by-product of haemoglobin digestion in the blood stages, and is carried over into the ookinete from the blood stage female gametocyte. In *P. berghei*, haemozoin-containing vacuoles accumulate around the edges of the crystalloid inclusion bodies (Fig. 4B and C) (Garnham *et al*., 1969; Sinden *et al*., 1985). This indicated that they could correspond to the crystalloids. Moreover, the fluorescent spots observed in ookinetes fitted the profile of crystalloids with respect to size and number per cell (Garnham *et al*., 1969). Indeed, immunoelectron microscopy of mCherry/PbSR/EGFP ookinetes exclusively labelled crystalloids (Fig. 4C), while crystalloids of wild-type ookinetes did not label (Fig. 4D), confirming that PbSR localizes to the crystalloid structures.

### PbSR loss-of-function phenotype

**Fig. 5:**
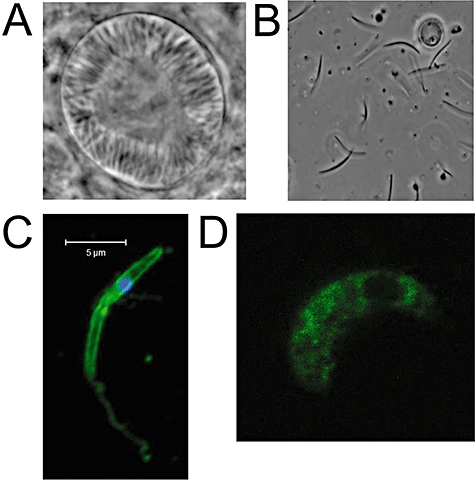
PbSR loss-of-function phenotype. A. Bright-field image of a sporulating ΔSRCR/EGFP oocyst at 15 days post infection. B. Midgut-associated sporozoites released from ΔSRCR/EGFP oocysts. C. Immunofluorescence image of a ΔSRCR/EGFP sporozoite with anti-CS antibodies (3D11), showing surface localization and trailing. D. Typical localization of PbSR in a ΔSRCR/EGFP ookinete, clearly showing the absence of focal spots.

We generated parasite line ΔSRCR/EGFP, in which the two central SRCR domains had been removed from the GFP-tagged PbSR-coding sequence (Fig. 1A). This parasite formed normal numbers of oocysts in mosquitoes, but oocyst sporulation was markedly impaired. In samples of pooled oocyst-infected guts, gently homogenized to release sporozoites, we failed to detect any sporozoites (compared with > 10 000 sporozoites per gut in control groups with comparable oocyst numbers), although we occasionally observed a sporulating oocyst by light microscopy. These appeared normal (Fig. 5A) and released sporozoites when subjected to gentle pressure (Fig. 5B). Moreover, these sporozoites displayed normal CS expression on their surface, and left CS reactive trails (Fig. 5C), indicating they possessed gliding motility. Nonetheless, in several infection experiments we failed to detect sporozoites in salivary glands, and were unable to successfully transmit parasites to naïve mice by infected mosquito bites (data not shown). To assess whether the reduced level of sporulation observed in the ΔSRCR/EGFP parasites was perhaps the result of a reduced, rather than abolished, level of functionality of the modified PbSR protein, we generated parasite line ΔPbSR/EGFP, in which the entire coding sequence for the mature PbSR protein had been structurally removed (Fig. 1). This parasite behaved in the same way as parasite line ΔSRCR/EGFP, supporting sporulation in a small fraction of oocysts (data not shown). Sporozoites were not detected in salivary glands, nor were these parasites successfully transmitted by infected mosquito bites. The combined results show that the central SRCR domains of PbSR are necessary for normal function of the protein. In addition, they show that PbSR is not essential for sporozoite formation in mosquitoes as reported previously (Claudianos *et al*., 2002), but that without functional PbSR sporulation levels are highly reduced.

GFP fluorescence was observed in female gametocytes of parasite line ΔSRCR/EGFP, indicating that the modified PbSR protein was normally expressed. Its localization in gametocytes appeared similar to that in parasites lines PbSR/EGFP and mCherry/PbSR/EGFP (data not shown). However, ookinetes of parasite line ΔSRCR/EGFP lacked the focal spots corresponding to the crystalloid inclusions and instead displayed dispersed green fluorescence (Fig. 5D), indicating that the mutated protein was no longer targeted to the crystalloids, or that the formation of crystalloids was affected in these parasites. The same dispersed GFP fluorescence pattern was observed in ookinetes of an independent clone of parasite line ΔSRCR/EGFP (Fig. S1), corroborating that this is the result of the parasites expressing modified PbSR. When carefully examining thin sections of ΔSRCR/EGFP ookinetes by electron microscopy, we could no longer find any crystalloid inclusions (0 positive in 150 independent ΔSRCR/EGFP ookinetes examined, compared with 47 positive in 150 independent mCherry/PbSR/EGFP ookinetes examined; *P* = 1.95e-16). Similarly, no crystalloids were found when we examined ookinetes of parasite line ΔPbSR/EGFP. These combined results indicate that PbSR is involved in the formation of the crystalloids. Pigment formation was not affected in parasite lines ΔSRCR/EGFP and ΔPbSR/EGFP (data not shown).

### *In vitro* oocyst culture

**Fig. 6:**
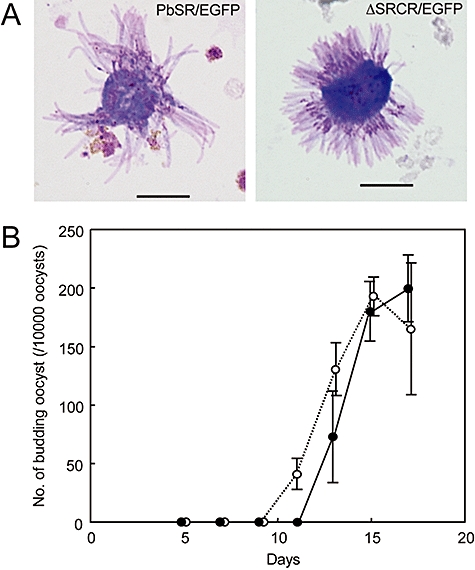
Oocyst development *in vitro*. A. Giemsa-stained sporulating oocysts of parasite lines PbSR/EGFP and ΔSRCR/EGFP. Scale bars represent 10 μm. B. Time-course of oocyst sporulation for PbSR/EGFP (dotted line) and ΔSRCR/EGFP (solid line). Each count was performed in triplicate and data points represents mean ± SD.

We assessed oocyst development *in vitro* using purified *in vitro* cultured ookinetes as starting material. In parasite line PbSR/EGFP, the fluorescent spots observed in ookinetes – corresponding to the crystalloids – typically disappeared within a day of ookinete-to-oocyst transition, which is in agreement with the observations of oocysts in infected mosquitoes. *In vitro* sporulation of ΔSRCR/EGFP oocysts was readily observed (Fig. 6A). In contrast to mosquito infections, this occurred at a comparable rate to that of parasite line PbSR/EGFP (Fig. 6B). These results confirm that PbSR is not essential for oocyst sporulation *per se*, and indicate that the function of PbSR could involve mosquito factors.

## Discussion

In this study we have generated and analysed genetically modified *P. berghei* parasites stably expressing single and dual fluorescent protein-tagged versions of PbSR. These parasite lines were used to assess PbSR protein expression and subcellular localization throughout the life cycle using western blot analysis and fluorescence microscopy. The results show that PbSR is most abundantly expressed in blood-stage macrogametocytes. PbSR is also detected in downstream macrogametes, zygotes, ookinetes and young oocysts, but not in sporozoites. Transcript analysis has previously shown that *pbsr*-specific mRNA is present in all life stages (Claudianos *et al*., 2002) and indeed proteomic analysis detected the protein in all life stages (Hall *et al*., 2005). Assuming that these are not false positive results, we have to consider the possibility that PbSR could be present in some life stages at protein concentrations below those detectable by our western blot and fluorescence assays.

Our PbSR expression data disagree with previous studies that detected PbSR in sporozoites using indirect immunofluorescence (Claudianos *et al*., 2002; Trueman *et al*., 2004). Recognizing that fluorescent protein tagging is less prone to artefacts than immunofluorescence, we infer that the likely cause of these discrepancies is due to non-specific cross-reactivity of the antibodies used in the earlier studies. Our expression data of PbSR are supported by a recent study that used genetic crosses of PbSR knockout parasites with either female-deficient or male-deficient parasites (Raine *et al*., 2007). This study showed that the lethal phenotype is only rescued when the wild-type *pbsr* allele is inherited from a female gametocyte, demonstrating that the function of PbSR is critical before expression of the protein from the male-derived allele can occur (i.e. before macrogamete fertilization). This observation is consistent with a scenario in which the macrogametocyte is the only life stage where discernible PbSR protein synthesis occurs, which fits well with our PbSR expression data (Fig. 2B). The expression profile for PbSR reported here also fits well with that of its human malaria *Plasmodium falciparum* orthologue PfCCP3 (also called *P*SLAP), which is reportedly present in gametocytes, but absent from late oocysts and sporozoites (Delrieu *et al*., 2002; Pradel *et al*., 2004).

Previous studies have pointed to the oocyst as the likely site of action for both the crystalloids and PbSR (Garnham *et al*., 1969; Claudianos *et al*., 2002). The data presented in this article are the first to demonstrate a functional link between PbSR and the crystalloids. Crystalloids develop via the ER/Golgi (Garnham *et al*., 1969; Davies, 1974; Mehlhorn *et al*., 1980). This fits with the presence of a typical N-terminal ER signal peptide in the translated gene sequence of *pbsr* (Claudianos *et al*., 2002), and the observed disulphide bond formation of PbSR reported here (Fig. 2A), both of which are strong indicators that the protein initially enters the secretory pathway. However, rather than exiting the parasite, our data show that PbSR remains intracellular and ends up associated with the crystalloids. The reason for this intricate mechanism of protein trafficking is unclear, but it could be a way for PbSR to be delivered to the oocyst in a non-active form. At a specific time after oocyst transition, PbSR could be activated/released to provide an optimal amount of material for interaction, for maximal effect. It is tempting to speculate that this time of activation would coincide with the loss of the crystalloids. By analogy, yolk proteins in insects have been shown to form crystal structures during oogenesis (Papassideri *et al*., 2007). These yolk proteins accomplish their roles as nutrients following specific proteolytic cleavage during the early stages of embryogenesis.

Oocyst development *in vitro* of our PbSR functional knockout parasite displayed normal sporulation rates (Fig. 6), demonstrating that PbSR is not essential for sporozoite development *per se*. This contrasts starkly with the rate of sporulation observed in parasite-infected mosquitoes, and suggests that the function of PbSR could involve mosquito factors. Under our culture conditions, insect components are absent and are substituted for culture medium supplemented with vertebrate serum. Another notable difference *in vitro* is the apparent absence of oocyst capsule formation, which could facilitate the uptake of components from the environment. Conceivably, if PbSR were involved in the uptake of specific mosquito components, or in protecting the parasite against adverse effects of mosquito factors, it is plausible that the different conditions *in vitro* could render the role of PbSR less critical, resulting in the increased sporulation rates observed. Given the apparent strict requirement for PbSR synthesis in blood-stage gametocytes (Raine *et al*., 2007), it is also possible that PbSR could be involved in the recruitment of essential components from the vertebrate host that cannot be acquired once the parasite resides in the insect. These components could then be deposited in the ookinete in the form of crystalloid inclusions for use during oocyst maturation. Because in the *in vitro* system vertebrate components are provided throughout oocyst development in the form of serum, the requirement for functional PbSR may be diminished. In view of the *in vitro* experiments reported here it is worth mentioning that *pfccp3*-disrupted *P. falciparum* parasites give rise to a significant level of seemingly normal oocyst sporulation in mosquitoes, although the sporozoites formed appear unable to infect the salivary glands (Pradel *et al*., 2004). Although the low levels of sporozoites formed in our PbSR knockout parasites make it difficult to assess their infectivity, this raises the possibility that the differences between PbSR and PfCCP3 with respect to oocyst sporulation could be merely quantitative.

It is conceivable that the crystalloids contain other proteins besides PbSR, and possibly also non-protein components (Trefiak and Desser, 1973). PbSR is part of a conserved protein family composed of several modular proteins, each containing ER signal peptides and multiple adhesive-type domains (Claudianos *et al*., 2002; Lasonder *et al*., 2002; Dessens *et al*., 2004; Pradel *et al*., 2004; Trueman *et al*., 2004). In *P. berghei*, different family members were shown to have similar loss-of-function phenotypes to PbSR (Raine *et al*., 2007). In *P. falciparum* also, the loss-off-function phenotypes of PfCCP3 and family member PfCCP2 were shown to be very similar (Pradel *et al*., 2004). This raises the possibility that distinct family members could be acting in concert as a protein complex, which may be reflected by our observation of PbSR forming high-molecular-weight protein complexes (Fig. 2A). Determining the composition of these protein complexes, as well as that of the crystalloid inclusions, will be instrumental in unravelling the molecular mechanisms that underlie the formation and function of this enigmatic organelle. The genetically modified parasite lines described here will provide useful tools in such experiments.

Our data point to a central role for the crystalloid in mosquito-to-host transmission, and show that its formation requires a parasite protein that is abundantly, and perhaps exclusively, expressed in blood-stage macrogametocytes. The crystalloids could therefore prove to be useful new targets for malaria transmission blockade, with the potential to inhibit their formation while the parasite develops in the vertebrate host.

## Experimental procedures

### Parasite maintenance, culture and purification

*Plasmodium berghei* ANKA clone 234 parasites were maintained as cryopreserved stabilates or by mechanical blood passage and regular mosquito transmission. To purify gametocytes, white blood cells were removed from gametocytaemic blood by passage through CF11 columns and further purified by centrifugation (300 *g* for 30 min) through 48% Nycodenz cushions, followed by phosphate-buffered saline (PBS) washes. Ookinete cultures were set up overnight from gametocytaemic blood as previously described (Arai *et al*., 2001; Carter *et al*., 2003). After 18–20 h, ookinetes were purified via ice-cold 0.17 M ammonium chloride lysis and centrifugation at 800 *g* for 10 min, followed by PBS washes. Mosquito transmission assays were as previously described (Dessens *et al*., 1999; 2001; 2003). Oocyst culture was based on Al-Olayan *et al*. (2002) without insect cells and basement membrane matrix, as described (Carter *et al*., 2007).

### Constructs

#### pLP-DHFR/SR

A 750 bp fragment corresponding to the 3′UTR of the *pbsr* gene was PCR amplified with primers SR-IF-Nhe-F2 (AAATACAGAAGCTAGTTATGTAAAATATATATAATTGCGTATAA) and SR-IF-Xba-R (GGGCGGCCGCTCTAGCATATGCACAAATATACATACATATAA) and introduced into NheI/XbaI-digested pCHT1-KO (Dessens *et al*., 2001) via In-Fusion PCR cloning (BD Bioscienses) to give plasmid pSR-REP1. A 201 bp fragment containing the *loxP*-prokaryotic promoter cassette was PCR-amplified with primers pLP-DHFR-F2 (ATAGGGCGAATTGGGCTGCAGATAACTTCGTATAGCATACATTATACG) and pLP-DHFR-R (CTGCAGGCATGCAAGCTGATCAACGTCAGGTGGCACTTTTCG) from template provided in the Creator Acceptor Vector Construction Kit (BD Biosciences). This PCR product was introduced into KpnI/HindIII-digested pSR-REP1 via In-Fusion PCR cloning to give plasmid pLP-DHFR/SR. This plasmid acts as the acceptor vector for all PbSR constructs.

#### pDNR-EGFP

A 1.2 kb fragment containing the enhanced green fluorescent protein (EGFP)-coding sequence plus the downstream 3′UTR from the *P. berghei dhfr* gene was PCR-amplified with primers pDNR-EGFP-F (ACGAAGTTATCAGTCGACTTAAGCTTAGGGGCCCTCATGAGTAAAGGAGAAGAACTTTTCACTGGA) and pDNR-EGFP-R (CCAAACGAATGGTCTAGTAAGCTCGAAATTGAAGGAAAAAACATCATTTG) from plasmid pOB079 (a gift from O. Billker) and introduced into SalI/HindIII-digested pDNR-Dual (BD Biosciences) via In-Fusion PCR cloning to give plasmid pDNR-EGFP.

#### pDNR-PbSR/EGFP

A 4.5 kb fragment containing the coding sequence plus the upstream 5′UTR from the *pbsr* gene was PCR-amplified with primers pDNR-PbSR-F (ACGAAGTTATCAGTCGACGGTACCACTCTATATAAGTATTTTTCTTCATCTTCG), containing a unique 5′ KpnI site, and pDNR-PbSR-R (ATGAGGGCCCCTAAGCTTAAGCGTTTCAAAAAGGTAAATGA) from *P. berghei* gDNA and introduced into SalI/HindIII-digested pDNR-EGFP via In-Fusion PCR cloning to give plasmid pDNR-PbSR/EGFP.

#### pDNR-PbSR/EGFP and derivatives

One microgram of plasmid pDNR-PbSR/EGFP served as template in PCR using primers ΔSR-F (*ATGGAAAGTTAAGCTT*AGGGGCCCTCAT) and ΔSR-R (*AAGCTTAACTTTCCAT*ATACGTTCATAAAAAATTGC). These two primers were designed to contain a 16 bp overlap (italicized). After PCR amplification (1 cycle of 94° for 2 min; 5 cycles of 94°C for 30 s, 62°C for 8 min), the template DNA was digested with DpnI and the amplified DNA self-ligated via In-Fusion PCR cloning to give plasmid pDNR-ΔSR/EGFP. In this plasmid, the PbSR-coding sequence is removed with the exception of the N-terminal signal peptide. The same approach was used to create plasmid pDNR-ΔSRCR/EGFP with primers ΔSRCR-F (*AAAGTAATGCAAGTGC*AGAAGAGGAAGG) and ΔSRCR-R (*GCACTTGCATTACTTT*TTATATCATCAATAGATGTCAAATGA). This plasmid has the two tandem SRCR domains removed from the PbSR-coding sequence.

The same approach was used to create plasmid pDNR-PbSR/EGFP/XhoI with primers XhoI-F (*AAAGCTCGAGTGGTGC*AAAGCGAAAGTTTG) and XhoI-R (*CACCACTCGAGCTTTC*CATATACGTTCATAAAAAATTGC). This plasmid has an extra XhoI restriction site introduced directly downstream of the predicted N-terminal signal peptide. The RFP variant mCherry sequence (Shaner *et al*., 2004) was amplified from plasmid pRSET-B (a gift from Dr Theresa Ward) with primers mCherry-F (ATATGGAAAGCTCGAGATGGTGAGCAAGGGCG) and mCherry-R (CTTTGCACCACTCGAGCTTGTACAGCTCGTCCATGC), and introduced into XhoI-digested pDNR-PbSR/EGFP/XhoI by in-fusion PCR cloning to give plasmid pDNR-mCherry/PbSR/EGFP. This plasmid contains the mCherry module directly downstream of the predicted N-terminal signal peptide sequence of PbSR.

#### pLP-PbSR/EGFP and derivatives

The PbSR-specific inserts contained within pDNR-PbSR/EGFP, pDNR-ΔSR/EGFP, pDNR-ΔSRCR/EGFP and pDNR-mCherry/PbSR/EGFP were introduced into pLP-DHFR/SR via Cre-*loxP* site-specific recombination (BD Biosciences) to give the transfection vectors pLP-PbSR/EGFP, pLP-ΔSR/EGFP, pLP-ΔSRCR/EGFP and pLP-mCherry/PbSR/EGFP respectively.

### Generation and genomic analysis of genetically modified parasites

Parasite transfection, pyrimethamine selection and dilution cloning were performed as previously described (Waters *et al*., 1997). Prior to performing transfections, plasmid DNA was digested with KpnI and SacII to remove the vector backbone. Genomic DNA extraction and Southern blot were performed as previously described (Dessens *et al*., 1999). All clonal genetically modified parasite populations were checked for the absence of wild-type parasites by diagnostic PCR.

### Western blot analysis

Parasite samples were heated directly in SDS-PAGE loading buffer at 70°C for 10 min. Proteins, in the absence or presence of 1% reducing agent (Invitrogen), were fractionated by electrophoresis through NuPage 4–12% Bis-Tris precast gels (Invitrogen) and transferred to PVDF membrane (Invitrogen) according to the manufacturer's instruction. The equivalent of 50 000 parasites per sample were analysed unless otherwise stated. Membranes were blocked for non-specific binding in PBS supplemented with 0.1% Tween 20 and 5% skimmed milk for 1 h at room temperature. Primary antibodies [rabbit polyclonal antibody to RFP (Abcam, ab34764); mouse monoclonal antibody to GFP (Invitrogen 33–2600); or mouse monoclonal antibody to CS (3D11)] diluted 1:5000 were applied to the membrane for 1 h at room temperature. After washing, membranes were incubated for 1 h with secondary antibody [for GFP and CS: goat anti-mouse IgG, horseradish peroxidase (HRP)-conjugated (Invitrogen 81-6520); for RFP: goat anti-rabbit IgG, HRP-conjugated (Abcam, ab6721)] diluted 1:5000 for 1 h at room temperature. After washing, signal was detected by chemilluminescence (Pierce ECL Western blotting substrate).

### Confocal microscopy

Live or paraformaldehyde-fixed parasite samples were mounted in Vectashield containing DAPI. Images were captured on a Zeiss Axiovert 200M inverted microscope using Zeiss LSM 510 software.

### Transmission electron microscopy

Parasites were prepared for electron microscopy by overnight fixation in 2.5% glutaraldehyde/2.5% paraformaldehyde/0.1 M Na cacodylate buffer at 4°C. Samples were post-fixed with 1% osmium tetroxide/0.1 M Na cacodylate buffer, washed with buffer followed by MilliQ water, bloc stained with 3% aqueous uranyl acetate, dehydrated in ascending ethanol concentrations, rinsed briefly in propylene oxide, then embedded and polymerized in Taab epoxy resin. Ultrathin sections were cut and mounted on Pioloform-coated copper grids and stained with lead citrate. Immunogold labelling was carried out as described (McDonald *et al*., 1995) using rabbit polyclonal antibody to GFP (Abcam, ab6556) diluted 1:500 and goat anti-rabbit IgG 10 nm gold-conjugated (BB International) diluted 1:400. Samples were examined on a Jeol 1200EX Mark II transmission electron microscope and digital images recorded with a 1K 1.3M pixel High Sensitivity AMT Advantage ER-150 CCD camera system.

### Crystalloid counts and statistical analysis

For each parasite line examined, thin sections of 150 ookinetes were examined by transmission electron microscopy and scored for the absence or presence of crystalloid. Scores were statistically analysed with the Fisher's Exact test (two-tailed).

## Supplementary material

This material is available as part of the online article from: http://www.blackwell-synergy.com/doi/abs/10.1111/

j.1365-2958.2008.06254.x

(This link will take you to the article abstract).
