# Supplementary material for: PbSR is synthesized in macrogametocytes and involved in formation of the malaria crystalloids
Source: Mol Microbiol. 2008 Apr 29;68(6):1560–9. doi: 10.1111/j.1365-2958.2008.06254.x (PMC2615194; doi:10.1111/j.1365-2958.2008.06254.x)

Fig. S1 Analysis of a second independent clone of parasite line ΔSRCR/EGFP. **A:** GFP fluorescence in an ookinete, showing lack of focal spots corresponding to crystalloids. **B:** Molecular analysis by PCR. Lane 1: 1kb product obtained with primers DHFRCAS3' (TCGTGGGCTACGTCCCGCAC) and SR3'-R (CGCCTTCACGCTGATGT), confirming genomic integration of the selectable marker gene cassette in the *pbsr* locus. Lane 2: 2.2kb product obtained with primers SR1250 (CGGAATTTTCGATTATAGAAGG) and pDNR-PbSR-R (ATGAGGGCCCCTAAGCTTAAGCGTTTCAAAAAGGTAAATGA), confirming the presence of the modified *pbsr* locus (missing the sequence encoding the SRCR domains) and the absence of the wild-type *pbsr* locus (would give band of 2.9kb). Lane M: DNA size markers.

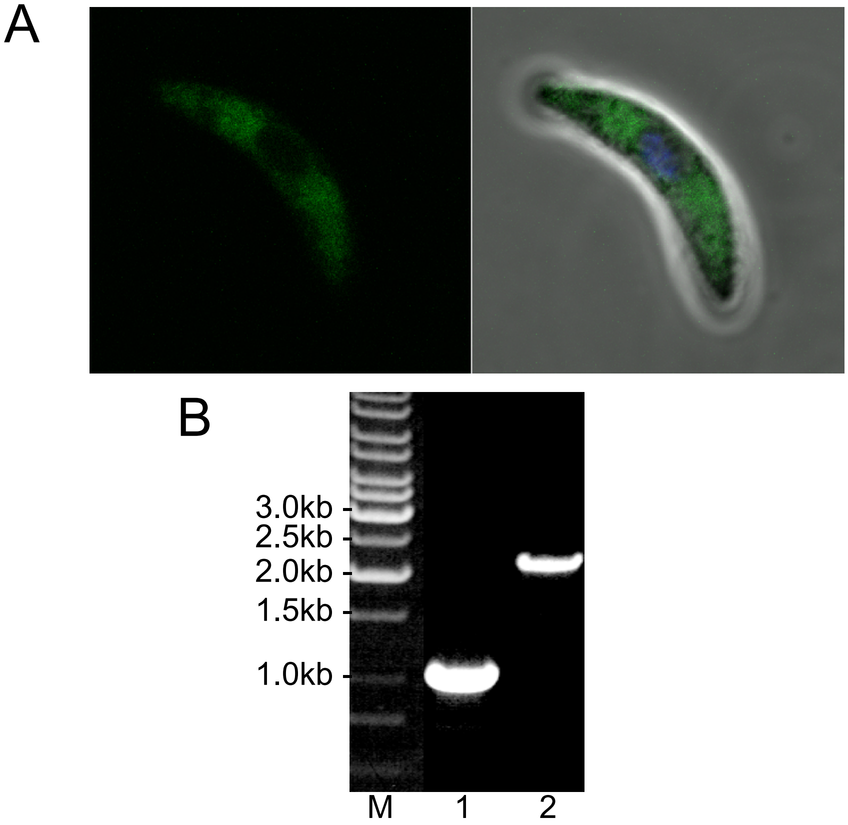

Supplement: Supplementary file 1 [file mmi0068-1560-SD1.pdf]
